# First‐Trimester Hematologic Inflammatory Markers and Pregnancy Loss in Women With Unexplained Recurrent Pregnancy Loss: A Retrospective Study of Associations and Discrimination

**DOI:** 10.1002/iid3.70535

**Published:** 2026-11-05

**Authors:** Ruifang Wang, Fangxiang Mu, Wei Zhang, Kexin Wang, Fang Wang

**Affiliations:** ^1^ Department of Reproductive Medicine Lanzhou University Second Hospital Lanzhou China; ^2^ Department of Obstetrics and Gynecology The First Affiliated Hospital, and College of Clinical Medicine of Henan University of Science and Technology Luoyang China

**Keywords:** neutrophil‐to‐lymphocyte ratio, pregnancy loss, systemic immune‐inflammation index, systemic inflammation, unexplained recurrent pregnancy loss

## Abstract

**Objective:**

This study aims to investigate whether complete blood count (CBC)‐derived systemic inflammation indices are associated with pregnancy loss in women with unexplained recurrent pregnancy loss (URPL).

**Methods:**

This retrospective observational study included 517 women with URPL who were recruited from Lanzhou University Second Hospital between September 2019 and December 2022. Participants were categorized into the pregnancy loss (*n* = 265) group and live birth (*n* = 252) group. CBCs obtained at 4–8 weeks of gestation were used to calculate the neutrophil‐to‐lymphocyte ratio (NLR), monocyte‐to‐lymphocyte ratio (MLR), platelet‐to‐lymphocyte ratio (PLR), systemic immune‐inflammation index (SII), and systemic inflammation response index (SIRI). We fitted multivariable logistic regression models adjusted for age, education, ethnicity, and body mass index. We assessed in‐sample discriminative performance using receiver operating characteristic (ROC) curves.

**Results:**

Compared with the live birth group, the pregnancy loss group had higher neutrophil counts, NLR, SII, SIRI, MLR, and PLR, and lower lymphocyte counts (all *p* < 0.001). After adjustment, higher NLR (OR = 3.05, 95% CI: 2.25–4.21), SIRI (OR = 2.74, 95% CI: 1.77–4.33), and SII (per 100‐unit increase, OR = 1.36, 95% CI: 1.24–1.51) were associated with greater odds of pregnancy loss. In tertile analyses, compared with the lowest tertile, the highest tertile was associated with higher odds of pregnancy loss for NLR (OR = 5.51), SII (OR = 2.88), SIRI (OR = 2.55), MLR (OR = 2.41), and PLR (OR = 3.41), with all *p*‐values for trend < 0.001. In ROC analyses, NLR showed the highest in‐sample discriminative performance (AUC = 0.752), followed by SII (AUC = 0.716).

**Conclusion:**

First‐trimester CBC‐derived inflammatory indices, particularly NLR and SII, were associated with pregnancy loss in women with URPL and may have potential discriminative value within this cohort. These findings require confirmation in prospective, multicenter studies with broader confounder assessment and external validation before any clinical application can be considered.

**Abbreviations**
AUC: area under the curve
BMI: body mass index
CBC: complete blood count
CI: confidence intervals
hCG: human chorionic gonadotropin
MLR: monocyte‐to‐lymphocyte ratio
NLR: neutrophil‐to‐lymphocyte ratio
OR: odds ratio
PLR: platelet‐to‐lymphocyte ratio
ROC: receiver operating characteristic
ROS: reactive oxygen species
RPL: recurrent pregnancy loss
SII: systemic immune‐inflammation index
SIRI: systemic inflammation response index
URPL: unexplained RPL

## 1 Introduction

Recurrent pregnancy loss (RPL), commonly defined as two or more consecutive pregnancy losses [1], is an important reproductive health problem affecting approximately 1%–5% of couples attempting conception [2, 3]. Despite standardized evaluation, no identifiable cause is found in up to half of cases, which are classified as unexplained RPL (URPL) [4]. URPL is associated with substantial psychological distress and clinical uncertainty regarding subsequent pregnancy outcomes [5, 6]. Increasing evidence suggests that immune dysregulation and systemic inflammation may contribute to pregnancy loss, as successful gestation requires tightly regulated maternal–fetal immune tolerance [7, 8], and disruption of this balance has been implicated in adverse pregnancy outcomes [9, 10].

In recent years, composite hematologic indices derived from complete blood count (CBC)—such as the systemic immune‐inflammation index (SII) and the systemic inflammation response index (SIRI)—have been investigated as markers of systemic inflammatory status. These indices integrate information across multiple leukocyte lineages and are available in routine clinical practice. Prior studies have linked higher first‐trimester SII and SIRI levels with gestational diabetes mellitus [11], and one report suggested that SIRI, but not neutrophil‐to‐lymphocyte ratio (NLR) or platelet‐to‐lymphocyte ratio (PLR), was higher among women with early pregnancy loss compared with term controls [12]. However, evidence regarding CBC‐derived inflammatory indices in women with URPL remains limited.

Therefore, we conducted a retrospective observational study of 517 women with URPL treated at the Lanzhou University Second Hospital between September 2019 and December 2022 to evaluate associations between first‐trimester CBC‐derived inflammatory indices (NLR, monocyte‐to‐lymphocyte ratio [MLR], PLR, SII, and SIRI) and pregnancy loss, and to assess their discriminative performance.

## 2 Materials and Methods

### 2.1 Study Design and Population

This retrospective study was conducted in the Department of Reproductive Medicine at the Lanzhou University Second Hospital from September 2019 to December 2022. Demographic characteristics, clinical features, laboratory results, and pregnancy outcomes of all patients were collected through outpatient medical records and the hospital information system. The Ethics Committee of the Lanzhou University Second Hospital waived the requirement for written informed consent given the retrospective and anonymous nature of the data analysis. This study was approved by the Medical Ethics Committee of the Lanzhou University Second Hospital (2023A‐584).

A total of 517 patients with URPL who attended the reproductive medicine outpatient clinic were included. At enrollment, gestational age was 4–8 weeks, and maternal age ranged from 18 to 46 years; all patients had documented outcomes for their current pregnancy. All included patients had a history of two or more unexplained miscarriages and no more than two live births. In addition, patients who met any of the following criteria were excluded: presence of high‐risk factors for miscarriage, such as chromosomal abnormalities (embryonic or maternal chromosomal abnormalities), anatomical abnormalities (e.g., uterine septum) [13, 14], autoimmune diseases (e.g., connective tissue diseases, systemic lupus erythematosus, rheumatoid arthritis), thrombotic diseases [15], endocrine factors (e.g., diabetes [16], polyendocrine metabolic ovarian syndrome, thyroid diseases), infectious factors (e.g., tuberculosis, rubella virus, syphilis, endometritis), and male factors (e.g., low sperm motility); pregnancy achieved through assisted reproductive technology; body mass index (BMI) > 28 kg/m^2^; and presence of other severe organ diseases (e.g., liver or kidney failure, thyroid cancer, breast cancer) (Figure 1). Patients with acute infections or hematological diseases were additionally excluded, as these conditions could affect hematological parameters.

**Figure 1:**
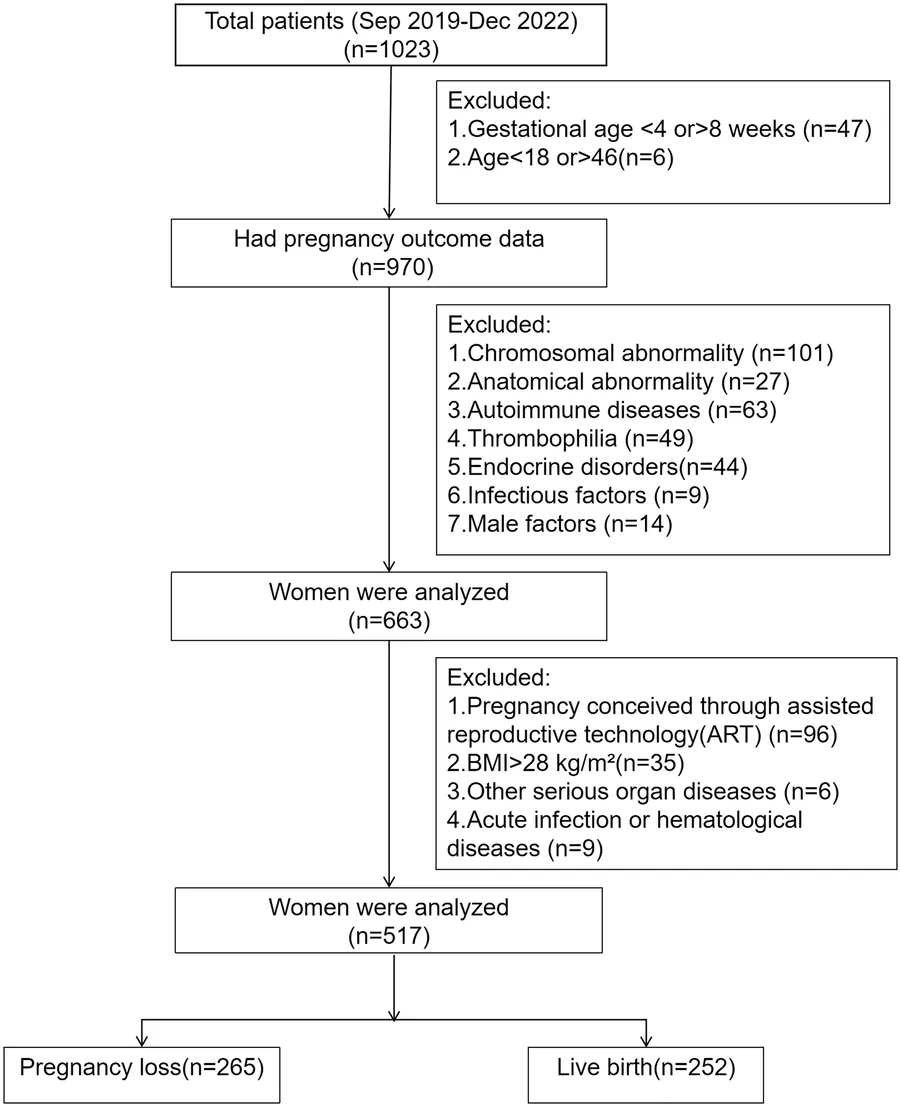
Flow chart of the population included in the final analysis.

As this was a retrospective study relying on medical records, data on therapeutic interventions during the pregnancy (e.g., progesterone supplementation, low‐molecular‐weight heparin, aspirin, or immunomodulatory therapies) were not consistently documented. They therefore could not be included in the analysis.

### 2.2 CBC Testing

All blood samples were collected during the patients’ first hospital visit at a gestational age of 4–8 weeks using 3 mL K_2_EDTA (dipotassium ethylenediaminetetraacetic acid) anticoagulant tubes. After collection, blood samples were gently inverted 5–8 times to ensure thorough mixing of blood with the anticoagulant, as recommended by the manufacturer. Samples were transported to the laboratory at ambient temperature (18°C–25°C) under protection from light and extreme temperatures. All CBC analyses were completed within 4 h of collection.

CBC analysis was performed using the Mindray BC‐6800 CRP automated hematology analyzer (Shenzhen Mindray Bio‐Medical Electronics Co. Ltd., Shenzhen, China). All hematological parameters were measured in the clinical laboratory of our hospital, which followed a comprehensive quality control program in accordance with the requirements of ISO 15189:2022. Internal Quality Control (IQC) was performed as follows: commercial quality control materials at three concentration levels (normal, low, and high) were analyzed daily before patient sample testing. The control materials were assayed at the beginning of each shift and after any maintenance or calibration procedures. Levey–Jennings charts were used to monitor the performance over time, and Westgard multi‐rules [1_3s_/2_2s_/R_4s_/41 s/1_2s_] were applied for run rejection criteria. The laboratory's coefficient of variation (CV) for each CBC parameter meets the minimum precision requirements based on biological variation and the Chinese health industry standard WS/T 406‐2012. External Quality Assessment (EQA) was performed as follows: the laboratory regularly participates in EQA/proficiency testing programs. These programs provide independent validation of the accuracy and comparability of our CBC results against peer laboratories. Satisfactory performance in all recent EQA cycles has been maintained, with all parameters falling within acceptable limits as defined by the program organizers.

NLR, MLR, PLR, SII (calculated as platelet count × neutrophil count/lymphocyte count), and SIRI (calculated as neutrophil count × monocyte count/lymphocyte count) were derived from the early pregnancy CBC results.

### 2.3 Pregnancy Outcomes

Pregnancy outcome data were obtained from face‐to‐face outpatient follow‐up and the hospital information system. Pregnancy loss was defined as biochemical pregnancy or clinical pregnancy loss occurring before 24 weeks of gestation. Clinical pregnancy loss was defined as the loss of an intrauterine pregnancy confirmed by ultrasonography or histology before 24 weeks of gestation. Biochemical pregnancy was defined as a positive urine or serum human chorionic gonadotropin (hCG) test without ultrasonographic evidence of an intrauterine gestational sac, followed by a spontaneous decline in hCG. Live birth was defined as the complete expulsion or extraction of a product of conception from its mother that, after separation, showed any evidence of life (e.g., breathing, heartbeat, umbilical cord pulsation, or definite movement of voluntary muscles), irrespective of gestational age [17]. Participants were categorized into two groups according to pregnancy outcome: pregnancy loss (*n* = 265) and live birth (*n* = 252).

### 2.4 Sample Size

The sample size was determined by the total number of patients available who met the inclusion and exclusion criteria. Statistical power analysis was performed for the binary outcome of “pregnancy loss” and “live birth” to estimate the minimum required sample size at a predefined significance level and power. Sample size calculation was conducted using the power analysis module for logistic regression in PASS software. For each primary variable (NLR, SII, and SIRI), calculations were performed using a pregnancy loss rate of P0 = 51.26% and odds ratios (OR) of 3.67, 1.44, and 3.64, respectively, with an assumed R^2^ = 0.2 for other covariates, a two‐sided α = 0.05, and a power = 0.9. The required sample sizes were calculated to be 31, 395, and 31, respectively. The final sample size of 517 exceeds the maximum required sample size, ensuring adequate statistical power for analysis.

### 2.5 Statistical Analysis

All analyses were performed using R (The R Foundation) and Empower Stats (X&Y Solutions, Inc., Boston, MA). Before analysis, the assumptions underlying all statistical methods were rigorously evaluated. Continuous variables were assessed for normality using the Shapiro–Wilk test (Table S1). Those meeting normality assumptions were presented as mean ± standard deviation and compared using Student's *t*‐test; variables violating normality assumptions were presented as median (interquartile range) and compared using the Mann–Whitney *U* test. Categorical variables were expressed as numbers (%) and compared using the Pearson chi‐square test when all expected cell counts were ≥ 5.

Before logistic regression analysis, multicollinearity was assessed using the variance inflation factor (VIF). The VIF test indicated that SIRI, SII, MLR, and PLR were inherently correlated because they are derived from shared CBC parameters, resulting in multicollinearity. Therefore, a hierarchical approach was adopted, with each inflammatory index entered individually into separate models rather than simultaneously, thereby avoiding collinearity in the estimation of regression coefficients. Logistic regression models were used to estimate ORs and 95% confidence intervals (CIs) for the associations between inflammatory indices (NLR, MLR, PLR, SII, and SIRI) and pregnancy loss, including Model 1 (adjusted for age), Model 2 (age, BMI, ethnicity/race and education), and Model 3 (age, BMI, ethnicity/race, education, age at menarche, age at first pregnancy, gravidity, parity, number of previous pregnancy losses, type of pregnancy loss, day of blood collection, and day of pregnancy termination). These variables represent baseline characteristics determined before inflammatory index measurement and are not mediators in the causal pathway; their inclusion minimizes confounding from clinically measurable non‐mediating factors, enabling more accurate estimation of the associations between inflammatory indices and pregnancy loss.

All indices were analyzed as continuous variables and by tertiles, with trend tests performed. Variables were confirmed to be ordinal and to meet logistic regression assumptions before tertile analysis, and Bonferroni correction was applied for multiple comparisons. Dose‐response relationships were evaluated using flexible curve fitting, with nonlinearity assessed when applicable, and further examined through threshold effect analyses to test for statistically significant inflection points and clarify whether dose‐response associations exhibited piecewise linear characteristics. Subgroup analyses were conducted by age, education level, ethnicity, and type of pregnancy loss; all subgroups met methodological assumptions and had sufficient sample sizes. Interactions were tested using cross‐product terms to avoid multicollinearity. Receiver operating characteristic (ROC) analysis was performed to evaluate the in‐sample discriminative performance of NLR, MLR, PLR, SII, and SIRI for distinguishing pregnancy loss from live birth within the study cohort. The ROC analyses were exploratory and not intended to establish or validate a prospective discriminative tool. All statistical tests were two‐sided, with *p* < 0.05 considered statistically significant.

## 3 Results

### 3.1 Baseline Characteristics and Inflammatory Indices

As shown in Table 1, the pregnancy loss group demonstrated significantly higher neutrophil counts, NLR, MLR, SII, SIRI, PLR, and an earlier day of pregnancy termination, but lower lymphocyte counts (all *p* < 0.001), and a lower proportion of women with college education or above (41.89% vs. 51.59%) compared to the live birth group; other demographic characteristics and routine hematological parameters did not differ significantly between the groups (all *p* > 0.05).

**Table 1:** Comparison of demographic features, laboratory results, and complete blood cell indices between the pregnancy loss group and live birth group.

Variables	Pregnancy loss group (*n* = 265)	Live birth group (*n* = 252)	*p* value
Maternal age (years) (mean ± SD)	31.06 ± 4.56	30.71 ± 4.30	0.370
Nation, *n* (%)			0.537
Han	232 (87.55)	225 (89.29)	
Other	33 (12.45)	27 (10.71)	
Education, *n* (%)			**0.027**
High school and below	154 (58.11)	122 (48.41)	
College and above	111 (41.89)	130 (51.59)	
BMI (kg/m^2^) (mean ± SD)	22.63 ± 2.62	22.29 ± 2.41	0.124
Gravidity (mean ± SD)	2.93 ± 1.22	2.89 ± 1.07	0.696
Parity (mean ± SD)	0.29 ± 0.51	0.32 ± 0.56	0.786
Previous pregnancy loss times (mean ± SD)	2.40 ± 0.83	2.34 ± 0.73	0.334
Type of pregnancy loss, *n* (%)			0.404
Primary	213 (80.38)	195 (77.38)	
Secondary	52 (19.62)	57 (22.62)	
Menarche age (years) (mean ± SD)	13.42 ± 1.25	13.41 ± 1.15	0.924
First pregnancy age (years) (mean ± SD)	26.89 ± 3.85	26.40 ± 3.65	0.142
Day of blood collection (days) (mean ± SD)	35.15 ± 5.95	35.82 ± 5.13	0.171
Day of pregnancy termination (days) (mean ± SD)	58.85 ± 4.52	269.61 ± 9.61	< 0.001
WBC (×10^9^/L) (mean ± SD)	7.92 ± 2.02	7.63 ± 1.95	0.095
RBC (×10^12^/L) (mean ± SD)	4.62 ± 0.39	4.57 ± 0.39	0.177
Hb (g/L) (mean ± SD)	139.46 ± 11.55	139.40 ± 14.09	0.955
Hct (L/L) (mean ± SD)	0.43 ± 0.04	0.42 ± 0.03	0.117
Platelet (×10^9^/L) (mean ± SD)	237.97 ± 55.44	235.64 ± 57.28	0.639
Pct (%) (mean ± SD)	0.24 ± 0.05	0.24 ± 0.05	0.258
Neutrophil (×10^9^/L) (mean ± SD)	5.83 ± 1.66	5.28 ± 1.53	**< 0.001**
Lymphocyte (×10^9^/L) (mean ± SD)	1.61 ± 0.48	1.86 ± 0.48	**< 0.001**
Monocyte (×10^9^/L) (mean ± SD)	0.37 ± 0.13	0.37 ± 0.12	0.968
MCV (fL) (mean ± SD)	91.19 ± 7.29	91.13 ± 5.79	0.911
PDW (%) (mean ± SD)	16.14 ± 0.71	16.14 ± 1.00	0.973
MPV (fL) (mean ± SD)	10.72 ± 1.57	10.64 ± 1.47	0.540
NLR (median [Q1, Q3])	3.51 (3.03, 4.29)	2.87 (2.37, 3.27)	**< 0.001**
MLR (median [Q1, Q3])	0.22 (0.18, 0.28)	0.20 (0.16, 0.24)	**< 0.001**
SII (median [Q1, Q3])	815.51 (656.27, 1072.41)	649.01 (521.53, 828.71)	**< 0.001**
SIRI (median [Q1, Q3])	1.28 (0.93, 1.68)	1.00 (0.77, 1.29)	**< 0.001**
PLR (median [Q1, Q3])	145.57 (117.26, 183.33)	127.40 (105.55, 150.00)	**< 0.001**

*Note:* Continuous variables with a normal distribution were compared using Student's *t*‐test; those with a non‐normal distribution were compared using the Mann–Whitney *U* test, and categorical variables were compared using the Pearson chi‐square test. All reported *p*‐values were derived from the corresponding statistical tests. Bold values indicate statically significant.

Abbreviations: BMI, body mass index; Hb, hemoglobin; Hct, hematocrit; MCV, mean corpuscular volume; MLR, monocyte to lymphocyte ratio; MPV, mean platelet volume; NLR, neutrophil to lymphocyte ratio; Pct, plateletcrit; PDW, platelet distribution width; PLR, platelet to lymphocyte ratio; RBC, red blood cell; SII, systemic immune‐inflammation index; SIRI, systemic inflammation response index; WBC, white blood cell.

### 3.2 Analysis of the Association Between Inflammatory Markers and Pregnancy Loss

In Model 1 (adjusted for age), NLR, SII, SIRI, MLR, and PLR were positively associated with pregnancy loss; these associations remained after further adjustment in Model 2 and Model 3. In Model 3, each 1‐unit increase in NLR was associated with 3.05‐fold higher odds of pregnancy loss (OR = 3.05, 95% CI: 2.25–4.21) (Table 2); each 100‐unit increase in SII was associated with a 36% increase in odds (OR = 1.36, 95% CI: 1.24–1.51); each 1‐unit increase in SIRI was associated with 2.74‐fold higher odds (OR = 2.74, 95% CI: 1.77–4.33); each 0.01‐unit increase in MLR was associated with a 5% increase in odds (OR = 1.05, 95% CI: 1.03–1.08); and each 1‐unit increase in PLR was associated with a 1% increase in odds (OR = 1.01, 95% CI: 1.01–1.02) (all *p* < 0.001). In Model 3, compared with the lowest tertile, participants in the highest tertile had higher odds of pregnancy loss for NLR (OR = 5.51), SII (OR = 2.88), SIRI (OR = 2.55), MLR (OR = 2.41), and PLR (OR = 3.41) (all *p* for trend < 0.001). After correction, the associations of NLR, SII, SIRI, MLR, and PLR with pregnancy loss remained statistically significant, indicating the robustness of these results. The dose‐response curves suggested monotonic increasing patterns, with potential nonlinearity particularly for PLR and SII (Figure 2). Threshold effect analyses showed no statistically significant threshold effects for PLR or SII (all *p* > 0.05; Table S2); thus, no clear inflection points were identified.

**Table 2:** The association of NLR, SII, SIRI, MLR, and PLR with pregnancy loss.

Variables	Model 1	*p* value	Model 2	*p* value	Model 3	*p* value	*Bonferroni p*
	OR (95% CI)		OR (95% CI)		OR (95% CI)		
NLR	3.40 (2.60, 4.55)	< 0.001	3.52 (2.68, 4.75)	< 0.001	3.05 (2.25, 4.21)	< 0.001	< 0.001
T1	ref		ref		ref		
T2	2.88 (1.85, 4.55)	< 0.001	3.02 (1.92, 4.79)	< 0.001	2.94 (1.84, 4.75)	< 0.001	
T3	8.12 (5.06, 13.28)	< 0.001	8.41 (5.18, 13.93)	< 0.001	5.51 (3.27, 9.46)	< 0.001	
*p* for trend		< 0.001		< 0.001		< 0.001	< 0.001
SII/100	1.44 (1.32, 1.58)	< 0.001	1.44 (1.32, 1.58)	< 0.001	1.36 (1.24, 1.51)	< 0.001	< 0.001
T1	ref		ref		ref		
T2	2.52 (1.63, 3.93)	< 0.001	2.48 (1.61, 3.89)	< 0.001	2.28 (1.44, 3.64)	< 0.001	
T3	4.53 (2.90, 7.18)	< 0.001	4.44 (2.82, 7.07)	< 0.001	2.88 (1.77, 4.74)	< 0.001	
*p* for trend		< 0.001		< 0.001		< 0.001	< 0.001
SIRI	3.56 (2.42, 5.38)	< 0.001	3.62 (2.43, 5.51)	< 0.001	2.74 (1.77, 4.33)	< 0.001	< 0.001
T1	ref		ref		ref		
T2	1.49 (0.97, 2.29)	0.069	1.58 (1.02, 2.46)	0.040	1.39 (0.88, 2.22)	0.161	
T3	3.44 (2.22, 5.39)	< 0.001	3.52 (2.25, 5.55)	< 0.001	2.55 (1.57, 4.16)	< 0.001	
*p* for trend		< 0.001		< 0.001		< 0.001	< 0.001
MLR ×100	1.06 (1.04,1.09)	< 0.001	1.06 (1.04, 1.09)	< 0.001	1.05 (1.03, 1.08)	< 0.001	< 0.001
T1	ref		ref		ref		
T2	1.04 (0.68, 1.60)	0.853	1.06 (0.69, 1.63)	0.799	1.02 (0.64, 1.62)	0.926	
T3	2.84 (1.84, 4.44)	< 0.001	2.88 (1.85, 4.51)	< 0.001	2.41 (1.50, 3.91)	< 0.001	
*p* for trend		< 0.001		< 0.001		< 0.001	< 0.001
PLR	1.01 (1.01, 1.02)	< 0.001	1.01 (1.01, 1.02)	< 0.001	1.01 (1.01, 1.02)	< 0.001	< 0.001
T1	ref		ref		ref		
T2	1.26 (0.82, 1.94)	0.286	1.33 (0.86, 2.05)	0.201	1.05 (0.66, 1.67)	0.850	
T3	2.97 (1.92, 4.64)	< 0.001	3.03 (1.95, 4.74)	< 0.001	3.41 (1.51, 3.88)	< 0.001	
*p* for trend		< 0.001		< 0.001		< 0.001	< 0.001

*Note:* Model 1: adjusted for age. Model 2: adjusted for age, BMI, ethnicity/race, education. Model 3: adjusted for age, BMI, ethnicity/race, education, age at menarche, age at first pregnancy, gravidity, parity, previous pregnancy loss times, type of pregnancy loss, day of blood collection, and day of pregnancy termination. SII was scaled per 100‐unit increase, and MLR was scaled per 100‐fold increase based on the magnitude of their original values. These transformations were applied to facilitate clinically interpretable effect estimates and standardized statistical reporting, and to avoid misleading or non‐informative results caused by excessively large or small original measurement units.

Abbreviations: BMI, body mass index; CI, confidence interval; MLR, monocyte to lymphocyte ratio; NLR, neutrophil to lymphocyte ratio; OR, odds ratio; PLR, platelet to lymphocyte ratio; SII, systemic immune‐inflammation index; SIRI, systemic inflammation response index.

**Figure 2:**
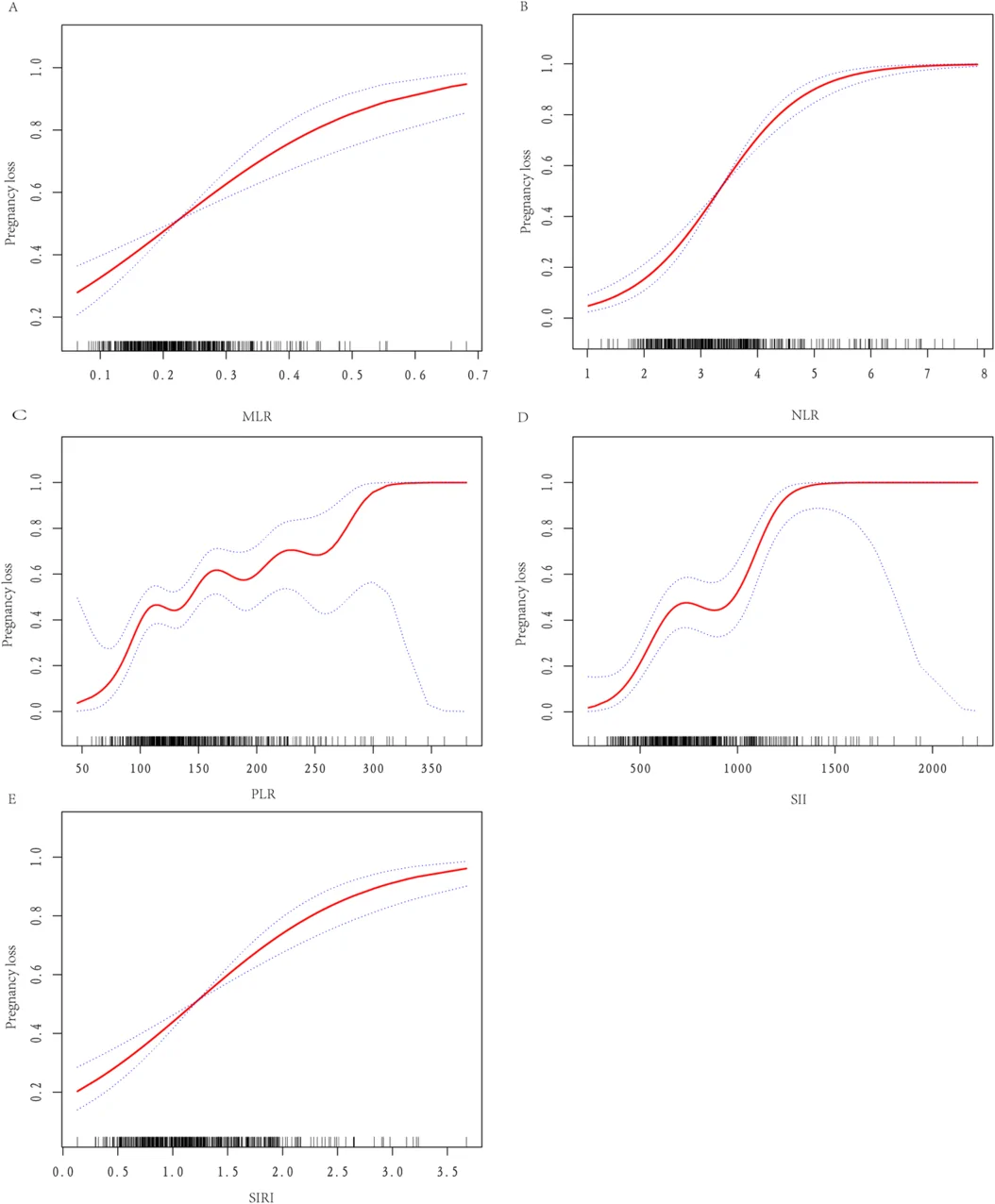
Associations between inflammatory markers and pregnancy loss. (A) Association between MLR and pregnancy loss. (B) Association between NLR and pregnancy loss. (C) Association between PLR and pregnancy loss. (D) Association between SII and pregnancy loss. (E) Association between SIRI and pregnancy loss. NLR, neutrophil to lymphocyte ratio; MLR, monocyte to lymphocyte ratio; SIRI, systemic inflammation response index; SII, systemic immune‐inflammation index; PLR, platelet to lymphocyte ratio.

### 3.3 Subgroup Analysis

We conducted stratified subgroup analyses by age, education level, ethnicity (Han vs. other ethnicities), and type of pregnancy loss, adjusting for all confounders (Table 3). For NLR, SII, SIRI, MLR, and PLR, no statistically significant interactions were detected across subgroups (all *p* for interaction > 0.05), indicating broadly consistent associations.

**Table 3:** Results of subgroup analysis.

Subgroups	OR (95% CI)	*p* value	*p* for interaction
**NLR**			
Age			0.493
< 35	3.27 (2.33, 4.71)	< 0.001	
≥ 35	2.90 (1.40, 6.87)	< 0.001	
Education			0.180
High school and below	3.86 (2.51, 6.21)	< 0.001	
College and above	2.87 (1.79, 4.81)	< 0.001	
Ethnicity			0.720
Han	2.98 (2.17, 4.20)	< 0.001	
Other	6.84 (1.94, 37.39)	0.009	
Type of pregnancy loss			0.412
Primary	2.90 (2.06, 4.19)	< 0.001	
Secondary	4.86 (2.28, 12.17)	< 0.001	
**SII**			
Age			0.810
< 35	1.37 (1.23, 1.53)	< 0.001	
≥ 35	1.46 (1.14, 1.95)	0.005	
Education			0.780
High school and below	1.40 (1.23, 1.61)	< 0.001	
College and above	1.37 (1.18, 1.61)	< 0.001	
Ethnicity			0.797
Han	1.37 (1.24, 1.53)	< 0.001	
Other	1.61 (1.13, 2.59)	0.021	
Type of pregnancy loss			0.874
Primary	1.37 (1.23, 1.55)	< 0.001	
Secondary	1.51 (1.20, 1.97)	0.001	
**SIRI**			
Age			0.489
< 35	2.53 (1.55, 4.23)	< 0.001	
≥ 35	5.64 (1.79, 22.10)	0.007	
Education			0.446
High school and below	2.39 (1.34, 4.38)	0.004	
College and above	4.22 (2.03, 9.32)	< 0.001	
Ethnicity			0.415
Han	2.57 (1.61, 4.22)	< 0.001	
Other	10.91 (1.65, 132.64)	0.028	
Type of pregnancy loss			0.294
Primary	2.41 (1.44, 4.11)	0.001	
Secondary	4.35 (1.67, 13.38)	0.005	
**MLR**			
Age			0.147
< 35	1.05 (1.02, 1.08)	0.001	
≥ 35	1.12 (1.04, 1.22)	0.005	
Education			0.323
High school and below	1.04 (1.01, 1.08)	0.027	
College and above	1.08 (1.04, 1.12)	< 0.001	
Ethnicity			0.736
Han	1.05 (1.02, 1.08)	< 0.001	
Other	1.13 (1.03, 1.27)	0.018	
Type of pregnancy loss			0.678
Primary	1.06 (1.02, 1.09)	< 0.001	
Secondary	1.04 (1.00, 1.10)	0.069	
**PLR**			
Age			0.993
< 35	1.01 (1.01, 1.02)	< 0.001	
≥ 35	1.01 (1.00, 1.02)	0.056	
Education			0.457
High school and below	1.01 (1.00, 1.02)	0.003	
College and above	1.01 (1.01, 1.02)	< 0.001	
Ethnicity			0.735
Han	1.01 (1.01, 1.02)	< 0.001	
Other	1.03 (1.01, 1.05)	0.020	
Type of pregnancy loss			0.075
Primary	1.01 (1.01, 1.02)	< 0.001	
Secondary	1.00 (0.99, 1.01)	0.419	

*Note:* All covariates were adjusted.

Abbreviations: CI, Confidence Interval; MLR: monocyte to lymphocyte ratio; NLR: neutrophil to lymphocyte ratio; OR, Odds ratio; PLR: platelet to lymphocyte ratio; SII, systemic immune‐inflammation index; SIRI, systemic inflammation response index.

### 3.4 ROC Analysis

Table 4 shows that the cutoff values derived from the ROC analyses for NLR, SII, SIRI, MLR, and PLR for discriminating between pregnancy loss and live birth were 3.19, 731.25, 1.22, 0.24, and 137.27, respectively. The corresponding area under the curves (AUCs) was 0.752, 0.716, 0.661, 0.622, and 0.642, all of which were statistically significant (*p* < 0.001). In terms of sensitivity and specificity, NLR showed a sensitivity of 64.91% and specificity of 71.83%, while SII showed a sensitivity of 63.77% and specificity of 66.67%, representing relatively balanced discriminative performance among the evaluated indices. Although MLR demonstrated a relatively high specificity (79.37%), its sensitivity was low (43.77%). PLR and SIRI exhibited moderate sensitivity (58.11% and 56.60%) and specificity (63.89% and 70.24%). These results indicate that the in‐sample discriminative performance of these inflammatory indices to distinguish pregnancy loss from live birth was limited.

**Table 4:** Summary of ROC analyses showing the cutoff values and discriminative performance of NLR, SII, SIRI, MLR, and PLR for distinguishing pregnancy loss from live birth among women with URPL.

Variable	Cut‐off	AUC	Sensitivity	Specificity	95% CI	*p* value
NLR	3.19	**0.752**	64.91%	71.83%	0.71–0.79	< 0.001
SII	731.25	**0.716**	63.77%	66.67%	0.67–0.76	< 0.001
SIRI	1.22	0.661	56.60%	70.24%	0.61–0.71	< 0.001
MLR	0.24	0.622	43.77%	79.37%	0.57–0.67	< 0.001
PLR	137.27	0.642	58.11%	63.89%	0.59–0.69	< 0.001

Abbreviations: MLR, monocyte to lymphocyte ratio; NLR, neutrophil to lymphocyte ratio; PLR, platelet to lymphocyte ratio; SII, systemic immune‐inflammation index; SIRI, systemic inflammation response index.

## 4 Discussion

This study evaluated associations between first‐trimester CBC‐derived inflammatory indices (NLR, SII, SIRI, MLR, and PLR) and pregnancy loss among women with URPL. Compared with the live birth group, the pregnancy loss group had higher neutrophil counts, NLR, MLR, SII, SIRI, and PLR, and lower lymphocyte counts, suggesting a more pro‐inflammatory hematologic profile in women with subsequent pregnancy loss.

In multivariable logistic regression model 3, higher NLR (OR = 3.05 per 1‐unit increase), SIRI (OR = 2.74 per 1‐unit increase), and SII (OR = 1.36 per 100‐unit increase) were associated with higher odds of pregnancy loss; tertile analyses showed increasing odds across higher categories. ROC analyses indicated acceptable discriminative performance for NLR (AUC = 0.752) and SII (AUC = 0.716), whereas other indices showed lower discriminative performance. These AUC values do not support stand‐alone clinical decision‐making. NLR and SII are best regarded as adjunctive risk markers alongside established clinical factors, and their clinical utility requires external validation, calibration, and demonstration of incremental value. Although these indices show modest discriminative performance within this cohort, their optimal thresholds remain to be determined.

The potential mechanisms underlying the observed associations between inflammatory markers and pregnancy loss warrant further consideration. It is increasingly recognized that immune imbalance and excessive inflammatory responses can contribute to implantation failure and disturbances in early placental development, both of which are implicated in RPL pathogenesis [18, 19]. Increased NLR, SII, SIRI, MLR, and PLR reflect relative neutrophilia or monocytosis and lymphopenia, indicating a shift toward a pro‐inflammatory state [20, 21]. Such an environment may trigger abnormal maternal immune activation, resulting in impaired trophoblast invasion, defective vascular remodeling at the maternal‐fetal interface, and ultimately compromising fetal viability. Additionally, aberrant platelet and neutrophil counts may promote a hypercoagulable state and microthrombus formation in placental vessels [22, 23], further impeding placental function and increasing the risk of pregnancy loss. For instance, NLR is widely recognized as an indicator of immune status, reflecting the balance between innate immune activation and adaptive immune tolerance [24, 25]. During inflammation, neutrophil and platelet counts typically increase while lymphocyte counts decrease, resulting in elevated NLR [20] and reflecting enhanced inflammatory activity. Notably, neutrophils can release reactive oxygen species (ROS) and promote pro‐inflammatory cytokine production such as IL‐6, IL‐8, and TNF‐α [26]. These mechanisms are consistent with our findings that NLR was significantly higher in URPL cases. Previous studies have also reported an association between elevated NLR and increased miscarriage risk [20]. However, a recent study of missed miscarriage found no NLR difference, perhaps reflecting differences in patient populations [27]. Nevertheless, these proposed mechanisms should be interpreted as biologically plausible explanations. Further translational and mechanistic studies are necessary to clarify these processes and to determine whether targeting systemic inflammation could offer therapeutic benefit for women at high risk of RPL.

The strengths of this study include the evaluation of multiple CBC‐derived inflammatory indices in a well‐defined URPL cohort and adjustment for key confounders, with discriminative performance assessed using ROC analyses. We also report cutoff values derived from ROC analyses in this cohort (e.g., NLR = 3.19, SII = 731.25) to inform future validation efforts. However, it should be noted that the inflammatory indices evaluated are mathematically and biologically interdependent, as they are derived from shared CBC components and thus represent different dimensions of the same underlying inflammatory state rather than independent biomarkers. Consequently, the observed associations should be interpreted as converging evidence for elevated systemic inflammation in pregnancy rather than multiple independent findings. Among these indices, NLR and SII demonstrated superior discriminative performance and may serve as more robust indicators for quantifying inflammatory status in this population, while the remaining indices provided supporting evidence for the observed associations. This study therefore does not treat these markers as independent sources of novel information, but rather aims to comprehensively evaluate the collective utility of CBC‐derived inflammatory indices for outcome discrimination in women with URPL.

Several limitations should be acknowledged. The retrospective design limits causal inference, and residual confounding from unmeasured factors—including therapeutic interventions, smoking, asymptomatic genital infections, and human papillomavirus infection [28]—cannot be ruled out. These unmeasured factors may have influenced both inflammatory indices and pregnancy outcomes. Additionally, the classification of URPL relied on retrospective medical records, so some misclassification is possible. We conducted multiple comparisons across regression models, stratified analyses, and ROC evaluations; although Bonferroni correction was applied to primary outcomes, the risk of type I error cannot be fully excluded. Most importantly, the reported cutoff values and discriminative performance were derived and evaluated in the same single‐center cohort without external validation. Together with the strict exclusion criteria, this limits generalizability to broader real‐world populations. Prospective multicenter studies with standardized sampling, diverse populations, and external validation are therefore warranted before these indices can be considered for any clinical application.

In conclusion, higher first‐trimester CBC‐derived inflammatory indices, particularly NLR and SII, were associated with pregnancy loss in this cohort of women with URPL and showed modest in‐sample discriminative performance. These indices should be interpreted as converging indicators of systemic inflammation rather than as independent biomarkers. Future prospective multicenter studies should standardize blood‐sampling protocols, externally validate clinically useful cutoff values in diverse populations, and assess whether NLR and SII provide incremental discriminative value beyond established clinical factors.

## Author Contributions

**Ruifang Wang:** conceptualization, methodology, writing – original draft. **Fangxiang Mu:** data curation, formal analysis, resources. **Wei Zhang:** software, supervision. **Kexin Wang:** validation, visualization. **Fang Wang:** conceptualization, writing – review and editing.

## Declaration of Generative AI Use

ChatGPT was used solely for language editing and grammar checking during manuscript preparation. All scientific content was reviewed and verified by the authors, who take full responsibility for the final manuscript.

## Ethics Statement

The Ethics Committee of the Lanzhou University Second Hospital agreed to waive the written informed consent due to the retrospective and anonymous nature of the data analysis. This study was approved by the Medical Ethics Committee of the Lanzhou University Second Hospital (2023A‐584).

## Consent

The authors have nothing to report.

## Conflicts of Interest

The authors declare no conflicts of interest.

## Supporting information


Supporting File 1

Supporting File 2

## Data Availability

The raw data supporting the conclusions of this article will be made available by the authors, without undue reservation.
