# Supplementary material for: First‐Trimester Hematologic Inflammatory Markers and Pregnancy Loss in Women With Unexplained Recurrent Pregnancy Loss: A Retrospective Study of Associations and Discrimination
Source: Immun Inflamm Dis. 2026 Nov 5;14(11):e70535. doi: 10.1002/iid3.70535 (PMC13737471; doi:10.1002/iid3.70535)
Supplement: Supplementary file 1 [file IID3-14-e70535-s001.docx]

**Table S1**. Normality test for continuous variables.

| **Variables** | **Shapiro-Wilk** | ***p* value** | **Skewness** |
| --- | --- | --- | --- |
| NLR (mean±SD) | 0.908 | <0.001 | 1.317 |
| MLR (mean±SD) | 0.905 | <0.001 | 1.507 |
| SII (mean±SD) | 0.916 | <0.001 | 1.359 |
| SIRI (mean±SD) | 0.933 | <0.001 | 1.148 |
| PLR (mean±SD) | 0.911 | <0.001 | 1.351 |

Abbreviations: NLR, neutrophil-to-lymphocyte ratio; MLR, monocyte-to-lymphocyte ratio; SIRI, systemic inflammation response index; SII, systemic immune-inflammation index; PLR, platelet-to-lymphocyte ratio.

**Table S2.** Threshold effect analysis.

| **Outcome** | **OR (95% CI)** | ***p* value** | **OR (95% CI)** | ***p* value** |
| --- | --- | --- | --- | --- |
| PLR |  |  | SII/100 |  |
| Model 1 |  |  |  |  |
| One line effect | 1.05 (1.02, 1.09) | <0.001 | 1.44 (1.32, 1.59) | <0.001 |
| Model 2 |  |  |  |  |
| Inflection point (K) | 104.73 |  | 5.72 |  |
| < K | 1.02 (1.01, 1.03) | 0.002 | 1.44 (1.32, 1.59) | <0.001 |
| > K | 1.01 (1.01, 1.02) | <0.001 | 1.45 (1.31, 1.60) | <0.001 |
| Log-likelihood ratio | 0.440 |  | 0.981 |  |

Abbreviations: OR, odds ratio; CI, confidence interval; SII, systemic immune-inflammation index; PLR, platelet-to-lymphocyte ratio.
